# Smartphone dependence and sport participation among Chinese adolescents: chain mediation by self-control and health beliefs and direct-path moderation by interpersonal support

**DOI:** 10.3389/fpsyg.2026.1876296

**Published:** 2026-07-01

**Authors:** Yuanye Hu, Zixuan Yang, Xingjun Zhou

**Affiliations:** Department of Leisure Services Sports, Pai Chai University, Daejeon, Republic of Korea

**Keywords:** adolescents, health beliefs, interpersonal support, self-control, smartphone dependence, sport participation

## Abstract

**Objective:**

This cross-sectional study examined the association between smartphone dependence and sport participation among Chinese adolescents and assessed whether self-control, health beliefs, and interpersonal support were statistically related to this association.

**Methods:**

A total of 1,610 students in Grades 5–9 completed self-report measures of smartphone dependence, sport participation, self-control, health beliefs, and interpersonal support. Structural equation modeling with bootstrapping was used to test a theoretically specified chain mediation model involving self-control and health beliefs. A separate simple moderation analysis using PROCESS Model 1 examined whether interpersonal support moderated the direct association between smartphone dependence and sport participation.

**Results:**

Smartphone dependence was negatively associated with sport participation. Bootstrapped structural equation modeling indicated three significant cross-sectional indirect associations: through self-control, through health beliefs, and through the theoretically specified sequence of self-control and health beliefs. In the separate moderation analysis, interpersonal support moderated the direct association between smartphone dependence and sport participation. The negative association was more pronounced among adolescents reporting higher interpersonal support, suggesting that general perceived support may not necessarily function as sport-specific support.

**Conclusion:**

The findings identify self-regulatory, health-cognitive, and social-contextual correlates of sport participation in the context of smartphone dependence. Given the cross-sectional design, the results should be interpreted as statistical associations rather than causal or temporal processes. They may inform future longitudinal and intervention studies examining whether self-regulatory skills, sport-related health beliefs, and action-oriented support can promote adolescent sport participation.

## 1 Introduction

### 1.1 Declining sport participation among adolescents: a public health concern

Regular sport participation is widely recognized as essential for the healthy physical, psychological, and social development of adolescents. Participation in sport and other forms of physical activity contributes to physical fitness, cardiometabolic health, bone health, motor and cognitive development, mental health, and overall well-being (Bengtsson et al., 2025; World Health Organization, 2024). Despite these benefits, insufficient physical activity among adolescents remains a major global public health concern. According to the World Health Organization, 81% of adolescents aged 11–17 years worldwide do not meet recommended physical activity levels, with adolescent girls being less active than boys (World Health Organization, 2024).

In China, recent evidence also indicates that physical activity and sport participation among children and adolescents remain insufficient. The China 2022 Report Card on Physical Activity for Children and Adolescents, based on a nationally representative sample of 133,006 school-aged children collected in 2020, rated overall physical activity as “C” and organized sport participation as “F,” concluding that physical activity levels among Chinese youth remain low and that most young people are below recommended guidelines (Liu et al., 2023). More recent empirical studies further support this concern. For example, a 2024 study of Chinese high school students found that only 30.9% met the recommendation of at least 60 min of daily moderate-to-vigorous physical activity, with girls reporting substantially lower compliance than boys (Xu et al., 2025). These findings suggest that sport participation among Chinese adolescents remains an important issue for public health, school education, and adolescent development.

This situation has prompted growing attention from educators, health professionals, and policymakers. Recent research indicates that Chinese adolescents’ sport participation is associated with multilevel factors, including environmental, organizational, interpersonal, and individual determinants (Ge et al., 2025). At the same time, digital media use has become an increasingly important behavioral context for adolescent health. Screen-based sedentary engagement may coexist or compete with active leisure, and longitudinal evidence from Chinese children and adolescents suggests that decreases in moderate-to-vigorous physical activity and increases in recreational screen time can persist over time and are linked to poorer mental health outcomes (Liu et al., 2024). These patterns raise important questions about how smartphone dependence is associated with adolescent sport participation and which psychological and social-contextual factors may help account for this association.

### 1.2 Smartphone dependence as a behavioral correlate of sport participation

The pervasive integration of smartphones into adolescents’ daily lives has raised growing concerns regarding the relationship between digital engagement and health-related behaviors. While mobile technologies offer convenience and avenues for social interaction, excessive and compulsive smartphone use has been increasingly associated with various maladaptive outcomes, including emotional distress, cognitive fatigue, social withdrawal, and behavioral displacement. Smartphone dependence, conceptualized as a behavioral tendency marked by loss of control, withdrawal symptoms, avoidance, and reduced functioning, has emerged as a salient construct in adolescent behavioral research (Leung, 2008; Panova and Carbonell, 2018).

For adolescents, whose developmental period is marked by heightened sensitivity to reward and ongoing maturation of self-regulation, the immersive and easily accessible nature of smartphone use may be relevant to participation in more structured and effortful forms of leisure such as sport. Compared with unstructured screen-based activities, sport participation usually involves sustained physical engagement, routine commitment, and delayed gratification—conditions that may be less attractive for adolescents reporting higher levels of smartphone dependence (Kajitani et al., 2020). Several studies have found that higher levels of smartphone dependence are associated with lower frequency, intensity, and duration of sport involvement (Zhao et al., 2022). Beyond possible time-use competition, smartphone dependence may also be linked to psychological characteristics relevant to sport engagement, including motivation, self-discipline, and health-related cognition.

These patterns suggest that smartphone dependence is not merely a technology-use variable, but also a behavioral correlate that may be meaningfully related to adolescents’ participation in health-promoting activities such as sport. However, the association between smartphone dependence and sport participation is unlikely to be reflected only in a single direct association. A more comprehensive understanding requires attention to psychological correlates and social-contextual conditions that may be associated with both digital behavior and sport participation, particularly within adolescent populations, where sport participation is developmentally significant and digital media use is highly embedded in daily life.

### 1.3 Indirect psychological associations: the roles of self-control and health beliefs

Understanding the association between smartphone dependence and adolescents’ sport participation requires attention to internal psychological factors that may help account for this relationship. Two conceptually distinct yet behaviorally connected constructs—self-control and health beliefs—are particularly relevant in this context, as they represent different levels of psychological functioning involved in health-related behavior. Self-control reflects a broader regulatory capacity to inhibit immediately rewarding behavior and maintain goal-directed action, whereas health beliefs reflect more proximal cognitive-motivational evaluations of sport participation, including perceived health relevance, perceived benefits, and confidence in one’s ability to engage in health-promoting behavior.

Self-control refers to an individual’s capacity to override impulsive tendencies and align actions with long-term goals or normative standards. According to self-control theory (Baumeister et al., 1998), this capacity plays a central role in managing conflicts between immediate gratification and future-oriented outcomes. Evidence from young Chinese samples suggests that smartphone dependence is associated with poorer self-regulatory functioning and lower engagement in physical activity (Wang et al., 2024; Zhang et al., 2024). Although these findings are not based exclusively on early-adolescent samples, they provide relevant evidence that excessive smartphone use may be linked to difficulties in self-monitoring, impulse control, and goal-directed behavior. In the present adolescent context, lower self-control may be associated with lower participation in structured and effortful activities such as regular sport participation, which often requires planning, persistence, and the ability to resist competing sedentary or screen-based alternatives.

Health beliefs, by contrast, reflect the cognitive and motivational evaluations individuals make regarding health-related behaviors. Grounded in the health belief model (HBM) (Rosenstock, 1974), this construct includes perceived susceptibility to health risks, perceived severity of negative outcomes, perceived benefits of protective behavior, and confidence in one’s ability to act, namely self-efficacy (Sheng et al., 2023). Adolescents with weaker health beliefs may be less likely to perceive sport participation as personally meaningful, beneficial, or necessary (Rostamian and Kazemi, 2016). Smartphone dependence may be negatively associated with sport-related health beliefs when adolescents are more oriented toward passive digital engagement, low-effort entertainment, or limited attention to health-related information. Conversely, adolescents who perceive sport as relevant to health protection, vitality, and self-development may report stronger involvement in sport despite competing leisure options.

Self-control and health beliefs were therefore selected because they capture two complementary psychological dimensions of adolescent sport participation: the capacity to regulate behavior and the cognition that gives health behavior personal meaning. They may operate as parallel psychological correlates because both are associated with smartphone dependence and sport participation through different processes. At the same time, they may also be connected sequentially. Self-control represents a relatively broad self-regulatory capacity that may support attention to long-term health goals, engagement with health-related information, and maintenance of value-guided behavior. Health beliefs are positioned more proximally to sport participation because they reflect adolescents’ evaluations of the necessity, benefits, and feasibility of engaging in sport. From this perspective, adolescents reporting lower self-control may be less likely to maintain strong sport-related health beliefs, which may in turn be associated with lower sport participation.

Together, self-control and health beliefs provide a useful psychological framework for examining the association between smartphone dependence and sport participation. Self-control reflects volitional capacity, whereas health beliefs capture health-related cognition and motivation. Considering both variables allows the present study to examine whether smartphone dependence is associated with sport participation through parallel and theoretically specified sequential indirect associations involving self-regulatory and health-cognitive factors. Because the present study used a cross-sectional design, this sequence should be understood as a theory-informed statistical specification rather than evidence of temporal ordering.

### 1.4 Social contexts matter: interpersonal support as a moderator

Adolescents’ sport participation does not occur in a psychological vacuum, but is embedded within a broader social ecology that includes family members, peers, teachers, and other significant others. Among the most widely studied contextual resources is interpersonal support (IPS), which refers to the perceived availability and quality of emotional, informational, or instrumental assistance from one’s social network (Shi et al., 2025). Drawing on social support theory, IPS is often conceptualized as a psychosocial resource associated with resilience, positive health behavior, and adaptation to stressors (Cohen and Wills, 1985).

In the context of adolescent sport behavior, interpersonal support may be relevant to sport participation by providing encouragement, companionship, information, and opportunities for engagement. Support from parents and peers has been shown to be associated with higher sport participation by enhancing perceived competence, increasing opportunities for engagement, and providing encouragement in the face of obstacles (Pluta et al., 2020; Gao et al., 2024). Teacher support may also be important in school-based sport participation because teachers can provide guidance on healthy digital engagement, physical education participation, outdoor activity opportunities, and structured encouragement for active lifestyles (Pluta et al., 2020). At the same time, adolescents with stronger support systems may be more receptive to external regulation and value reinforcement, which are particularly important during early stages of sport habit formation (Li et al., 2025).

The role of interpersonal support may be especially important when considering behavioral tendencies such as smartphone dependence. Some adolescents with higher smartphone dependence may report lower sport participation, whereas others may maintain active lifestyles within supportive family, peer, or school contexts. However, interpersonal support may not operate in a uniformly protective way. It may function as a buffering factor, or alternatively as a conditional factor whose meaning depends on adolescents’ level of smartphone dependence and the context in which support is experienced (Yetim et al., 2025). For example, support that is closely connected to digital communication may coexist with frequent smartphone-based social interaction, whereas support that is specifically oriented toward physical activity may be more directly related to sport participation. Clarifying the direction and magnitude of this moderating pattern is therefore important for understanding how social-contextual factors are related to the association between smartphone dependence and sport participation.

By examining interpersonal support as a moderator of the direct association between smartphone dependence and sport participation, the present study situates adolescents’ sport participation within a socio-developmental context and extends the model beyond intrapersonal psychological factors. This approach allows for a more nuanced examination of whether the association between smartphone dependence and sport participation differs across adolescents with varying levels of perceived interpersonal support, without assuming that interpersonal support necessarily conditions the indirect associations involving self-control and health beliefs.

### 1.5 The present study

Although prior studies have examined smartphone dependence and adolescents’ health-related behaviors, several conceptual and empirical gaps remain. First, existing research has often focused on direct associations between smartphone dependence and physical activity or sport participation, with less attention to the psychological factors that may help account for this relationship. In particular, limited research has examined whether self-control and health beliefs operate together as parallel and sequential psychological correlates in the association between smartphone dependence and sport participation. Second, although social support has been widely discussed in relation to adolescent health behavior, relatively little is known about whether interpersonal support conditions the direct association between smartphone dependence and sport participation.

To address these gaps, the present study examined a chain mediation and direct-path moderation model among Chinese adolescents. Specifically, the study investigated whether smartphone dependence was associated with sport participation directly and indirectly through self-control and health beliefs. In addition, the study examined whether interpersonal support moderated the direct association between smartphone dependence and sport participation. The model focused on statistical mediation and direct-path moderation rather than conditional indirect effects. Given the cross-sectional design, the proposed model was intended to test theory-informed statistical associations rather than causal or temporal processes.

Based on the above reasoning, the following hypotheses were proposed:

*H1*: Smartphone dependence would be negatively associated with sport participation.

*H2*: Self-control would statistically mediate the association between smartphone dependence and sport participation.

*H3*: Health beliefs would statistically mediate the association between smartphone dependence and sport participation.

*H4*: Smartphone dependence would be indirectly associated with sport participation through the theoretically specified sequence of self-control and health beliefs.

*H5*: Interpersonal support would moderate the direct association between smartphone dependence and sport participation.

**Figure 1:**
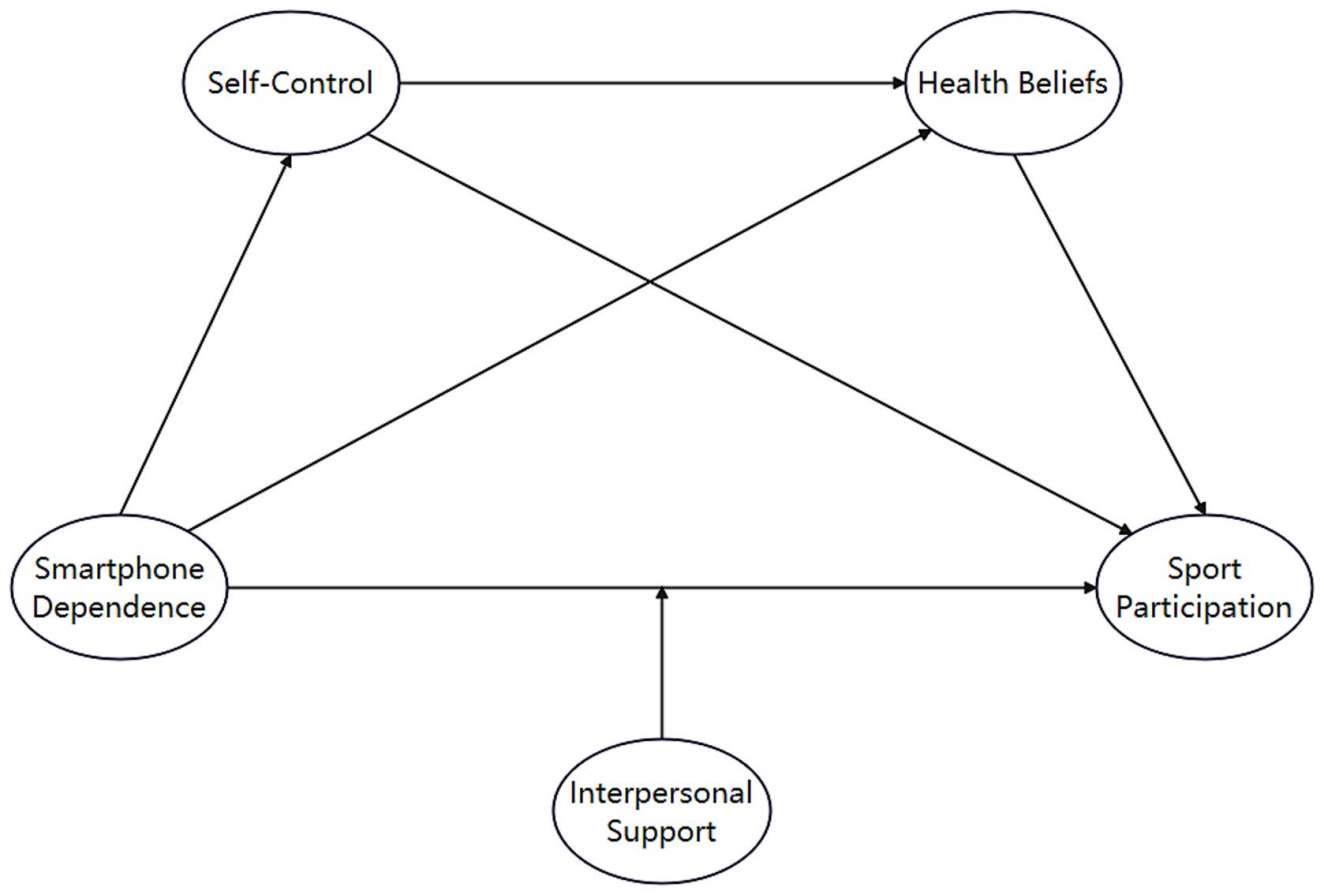
Conceptual model of the chain mediation and direct-path moderation analysis. The model specifies self-control and health beliefs as statistical mediators in the association between smartphone dependence and sport participation. Interpersonal support is specified as a moderator of the direct association between smartphone dependence and sport participation. Conditional indirect effects were not tested.

By integrating volitional, cognitive, and social-contextual factors into a unified framework, this study aimed to provide a more comprehensive understanding of the psychological correlates of adolescent sport participation in the context of smartphone dependence. The proposed conceptual model is presented in Figure 1.

## 2 Method

### 2.1 Participants and procedure

A cross-sectional survey was conducted among students from Grades 5 to 9 in general primary and secondary schools in Shandong Province, China. Data were collected from approximately 40 schools located in four prefecture-level cities: Jinan, Zibo, Qingdao, and Linyi. A school-based sampling procedure combining stratification and convenience recruitment was adopted. Grade level and school location were used as the main stratification criteria to ensure that the sample included students from both primary and secondary school stages as well as from urban and rural school contexts. Within these predefined strata, participating schools and classes were selected based on feasibility, school willingness, and administrative cooperation. Therefore, the sample should be interpreted as a stratified school-based convenience sample rather than a nationally representative random sample.

Although the research team was affiliated with Pai Chai University in the Republic of Korea, data collection was conducted in China with the cooperation and permission of the participating schools. This study was reviewed and approved by the Ethics Committee of the Department of Sports and Leisure Services, Pai Chai University, Republic of Korea. All research procedures were conducted in accordance with the ethical requirements of Pai Chai University, relevant local regulations and school administrative requirements in China, and the ethical principles of the Declaration of Helsinki.

Prior to data collection, permission was obtained from the participating schools. Written informed consent was obtained from the legal guardians of all adolescent participants, and assent was obtained from the participants themselves. Participation was voluntary, and participants were informed that they could withdraw from the study at any time. All questionnaires were completed anonymously during regular school hours with the support of trained research assistants and cooperating school personnel. All data were anonymized prior to analysis to ensure confidentiality and to protect the rights and welfare of participants.

A total of 1,750 questionnaires were administered. After excluding invalid responses due to missing data, patterned answering, or logical inconsistencies, a total of 1,610 valid cases were retained, yielding a valid response rate of 92.00%. The final sample included 799 boys and 811 girls, covering students from Grades 5 to 9, with 803 rural students and 807 urban students. This distribution indicates that the sampling procedure achieved broad coverage across the predefined strata of gender, grade level, and school location.

To evaluate the adequacy of the sample size, two widely accepted criteria were applied: the item-based ratio and the variable-based ratio. The final survey instrument consisted of 69 items across 19 latent dimensions. According to the recommended 5:1 to 10:1 participant-to-item ratio (Suhr, 2006), the minimum required sample size would range from 345 to 690 participants. Additionally, applying the 10:1 to 20:1 participant-to-variable ratio for structural equation modeling (Kline, 2016), a minimum of 190 to 380 participants would suffice for 19 observed latent variables. The final valid sample of 1,610 adolescents substantially exceeded both thresholds, ensuring sufficient statistical power and stability of parameter estimation. Detailed demographic characteristics and descriptive statistics for study variables are presented in Table 1.

**Table 1:** Demographic characteristics of the sample (*n* = 1,610).

Basic information	Category	Frequency	Percentage
Gender	Male	799	49.63
	Female	811	50.37
Grade	Grade 5	323	20.06
	Grade 6	406	25.22
	Grade 7	417	25.90
	Grade 8	315	19.57
	Grade 9	149	9.25
School location	Rural	803	49.88
	Urban	807	50.12
Sport Type	Individual Sports	304	18.88
	Dual Sports	541	33.60
	Team Sports	765	47.52

### 2.2 Measures

Five core constructs were assessed using established or adapted self-report measures. Because some instruments were originally developed or validated in related Chinese youth or young adult populations, item wording and response formats were reviewed to improve age appropriateness for students in Grades 5–9 and to align the measures with the adolescent school context. For adapted measures, the goal was to preserve the original construct meaning while making the wording understandable for younger adolescents. No independent validation sample was used before the main survey; therefore, the psychometric performance of the measures was evaluated in the present sample using internal consistency reliability analysis and confirmatory factor analysis.

All measures used five-point response formats. Most psychological items were rated from 1 (“strongly disagree”) to 5 (“strongly agree”), whereas the sport participation items used ordered response categories appropriate to intensity, frequency, and duration. Scale scores were calculated by averaging item responses after reverse coding where applicable. Higher mean scores indicated higher levels of the corresponding construct.

Smartphone dependence (SPD) was measured using the 17-item mobile phone dependence scale developed by Leung (2008). The scale includes four dimensions: loss of control, withdrawal, avoidance, and inefficiency. A representative item assessed whether students spent more time on their smartphone than intended. Higher mean scores indicate a higher level of smartphone dependence. In the present study, the scale was used to assess adolescents’ dependence-related smartphone use tendencies in daily life.

Sport participation (SPT) was assessed using a 3-item measure adapted from Liang (1994), covering three aspects of physical activity participation: intensity, frequency, and duration. A representative item assessed how often students usually participated in sport or physical exercise. The items were adapted to capture adolescents’ general level of sport or physical exercise participation in school and leisure-time contexts. Higher mean scores indicate a higher level of sport participation.

Self-control (SCT) was measured using the revised Chinese Self-Control Scale developed by Tan and Guo (2008). The scale consists of 19 items and includes five subdimensions: impulse control, healthy habits, entertainment restraint, work focus, and resistance to temptation. A representative item assessed students’ ability to resist temptation. Items requiring reverse coding were reverse-scored before calculating the mean score, so that higher mean scores consistently indicated higher self-control. Because the original revision was validated among Chinese college students, its applicability to the present adolescent sample was further evaluated through reliability analysis and confirmatory factor analysis.

Health beliefs (HLB) were assessed using an 18-item measure developed with reference to the Health Belief Model and prior Chinese research on sport-related health beliefs among adolescents and youths (Rosenstock, 1974; Sun et al., 2024). The measure covered four dimensions: health consciousness, perceived susceptibility, perceived severity, and self-efficacy. A representative item assessed whether students believed that regular sport participation was important for maintaining health. The items were used to assess adolescents’ sport-related health cognition, including their perceived relevance, importance, and feasibility of engaging in sport participation. Higher mean scores indicate stronger sport-related health beliefs. The reliability and construct validity of this measure were examined in the current sample.

Interpersonal support (IPS) was measured using the Chinese version of the Perceived Social Support Scale revised by Jiang (1999). The scale consists of 12 items covering three dimensions: family support, friend/peer support, and support from significant others. A representative item assessed whether students perceived their family as willing to provide help. Although the original PSSS commonly uses a 7-point response format, a 5-point response format was adopted in this study for consistency with the other measures and to improve comprehensibility among younger participants. The adapted IPS measure assessed general perceived support from family members, friends/peers, and significant others. Teacher support was not assessed as a separate dimension in this scale, although some adolescents may have considered teachers when responding to items concerning significant others. Higher mean scores indicate higher perceived interpersonal support. The reliability and construct validity of the adapted version were re-examined in the present sample.

### 2.3 Data analysis strategy

Data analyses were conducted using SPSS 27.0, AMOS 26.0, and the PROCESS macro (Version 4.0). First, SPSS 27.0 was used to conduct descriptive statistics, internal consistency reliability analysis, and Pearson correlation analysis. Cronbach’s *α* coefficients of 0.70 or above were considered acceptable for internal consistency reliability (Tavakol and Dennick, 2011). Preliminary assessment of common method bias (CMB) was performed using Harman’s single-factor test, with 40% of the variance explained by the first unrotated factor used as the reference threshold (Podsakoff et al., 2003). Group differences across demographic variables were examined using independent-samples *t*-tests and one-way ANOVA, with statistical significance set at *p* < 0.05. Where appropriate, effect sizes were reported using Cohen’s d for *t*-tests and η^2^ for ANOVA (Field, 2013). For significant grade-level differences, *post hoc* comparisons with Bonferroni correction were conducted to identify which grade groups differed from one another.

Second, AMOS 26.0 was used to conduct confirmatory factor analysis (CFA) to evaluate the measurement properties of the study variables. The overall measurement model was first examined, and construct-specific CFA models were also estimated to provide measurement model fit information for each key construct where applicable. Measurement quality was evaluated using standardized factor loadings, composite reliability (CR), and average variance extracted (AVE). Standardized factor loadings of 0.50 or above, CR values of 0.70 or above, and AVE values of 0.50 or above were considered acceptable (Fornell and Larcker, 1981). Discriminant validity was assessed using the Fornell–Larcker criterion, whereby the square root of AVE for each construct should exceed its correlations with other constructs (Fornell and Larcker, 1981). CMB was further examined using a CFA-based one-factor model and a common latent factor model (Podsakoff et al., 2003). Model fit was evaluated using multiple indices, including χ^2^, degrees of freedom, χ^2^/df, CFI, TLI, RMSEA, and SRMR. The following criteria were used to indicate acceptable model fit: χ^2^/df < 5.00, CFI ≥ 0.90, TLI ≥ 0.90, RMSEA ≤ 0.08, and SRMR ≤ 0.08 (Kline, 2016).

Third, structural equation modeling (SEM) in AMOS 26.0 was applied to test the theoretically specified chain mediation model involving smartphone dependence, self-control, health beliefs, and sport participation. The significance of direct, indirect, and total associations was evaluated using bias-corrected bootstrapping with 5,000 resamples and 95% confidence intervals. Indirect associations were considered statistically significant when the 95% confidence interval did not include zero (Kline, 2016). Because the data were cross-sectional, the mediation results were interpreted as statistical indirect associations rather than evidence of causal or temporal processes.

Finally, moderation analysis was conducted using the PROCESS macro (Model 1) in SPSS to examine whether interpersonal support moderated the direct association between smartphone dependence and sport participation. The interaction effect was tested using bootstrapped confidence intervals based on 5,000 samples. When the interaction term was significant, simple slope analysis was conducted to interpret the association between smartphone dependence and sport participation at low and high levels of interpersonal support, defined as one standard deviation below and above the mean of the moderator (Hayes, 2018). The mediation and moderation analyses were conducted as complementary but separate analyses. Conditional indirect effects were not estimated; therefore, the moderation analysis should be interpreted as a test of direct-path moderation rather than moderated mediation.

Gender, grade level, school location, and sport type were examined in the preliminary group-difference analyses described above. These demographic variables were not entered as covariates in the SEM or PROCESS moderation model. Thus, the main model estimates represent the associations among the theoretical variables without demographic covariate adjustment.

## 3 Results

### 3.1 Descriptive statistics, reliability, measurement validity, and correlational analysis

As shown in Table 2, all five study variables demonstrated acceptable reliability and measurement quality. The mean scores ranged from 2.43 (SPD) to 3.59 (SCT), with standard deviations ranging from 0.69 to 0.80, indicating moderate dispersion across participants. Overall, adolescents reported a relatively low-to-moderate level of smartphone dependence (M = 2.43, SD = 0.76), whereas sport participation (M = 3.55, SD = 0.80), self-control (M = 3.59, SD = 0.69), and health beliefs (M = 3.56, SD = 0.78) were above the midpoint of the 5-point scale. Interpersonal support was close to the scale midpoint (M = 3.07, SD = 0.73). These descriptive patterns suggest that smartphone dependence was not extremely high on average in the present sample, while adolescents generally reported moderate-to-favorable levels of sport participation, self-regulation, and sport-related health beliefs.

**Table 2:** Descriptive statistics, internal consistency reliability, and measurement validity of study variables.

Variable	M	SD	Cronbach’s α (Range)	Std. factor loading	CR	AVE
SPD	2.43	0.76	0.940 (0.803–0.925)	0.699–0.821	0.846	0.579
SPT	3.55	0.80	0.901	0.654–0.833	0.760	0.517
SCT	3.59	0.69	0.958 (0.898–0.920)	0.736–0.772	0.874	0.581
HLB	3.56	0.78	0.917 (0.803–0.879)	0.789–0.810	0.876	0.639
IPS	3.07	0.73	0.902 (0.825–0.869)	0.800–0.823	0.794	0.659

SPD, smartphone dependence; SPT, sport participation; SCT, self-control; HLB, health beliefs; IPS, interpersonal support. M, mean; SD, standard deviation; Cronbach’s α (Range) reports the internal-consistency coefficient for the full scale, followed in parentheses by the minimum and maximum Cronbach’s α values across its first-order subscales; Std. factor loading, standardized factor-loading range obtained from the confirmatory factor analysis; CR, composite reliability; AVE, average variance extracted.

Cronbach’s *α* coefficients ranged from 0.901 (SPT) to 0.958 (SCT), indicating acceptable to excellent internal consistency reliability. For multidimensional constructs, the Cronbach’s α coefficients of the first-order subscales were also acceptable. Standardized factor loadings for each construct exceeded 0.65, supporting the adequacy of the item–construct relationships. Composite reliability (CR) values ranged from 0.760 to 0.876, exceeding the conventional 0.70 benchmark and indicating acceptable composite reliability. Average variance extracted (AVE) values ranged from 0.517 to 0.659, supporting convergent validity.

In addition, construct-specific CFA results further supported the measurement quality of the study variables. The smartphone dependence model showed good fit to the data (CFI = 0.975, TLI = 0.972, SRMR = 0.024, RMSEA = 0.040, 90% CI [0.037, 0.043]). The sport participation model also demonstrated acceptable fit (CFI = 0.992, TLI = 0.988, SRMR = 0.017, RMSEA = 0.042, 90% CI [0.033, 0.051]). The self-control model showed good fit (CFI = 0.978, TLI = 0.977, SRMR = 0.021, RMSEA = 0.027, 90% CI [0.025, 0.029]). The health beliefs model demonstrated good fit (CFI = 0.982, TLI = 0.977, SRMR = 0.027, RMSEA = 0.044, 90% CI [0.039, 0.049]). The interpersonal support model also showed excellent fit (CFI = 0.997, TLI = 0.994, SRMR = 0.011, RMSEA = 0.034, 90% CI [0.018, 0.051]). Taken together, the reliability, factor-loading, CR, AVE, and construct-specific CFA results supported the adequacy of the measures used in the present sample.

Table 3 reports the bivariate correlations among the study variables and the results of discriminant validity testing. Smartphone dependence (SPD) was negatively correlated with sport participation (SPT, *r* = −0.488), self-control (SCT, *r* = −0.472), health beliefs (HLB, *r* = −0.487), and interpersonal support (IPS, *r* = −0.243). In contrast, SPT showed significant positive associations with SCT (*r* = 0.556), HLB (*r* = 0.667), and IPS (*r* = 0.231). All correlations were significant at *p* < 0.001. Moreover, the square root of AVE for each construct, shown on the diagonal of Table 3, exceeded its correlations with other constructs, satisfying the Fornell–Larcker criterion and supporting discriminant validity.

**Table 3:** Correlations and assessment of discriminant validity.

Variable	SPD	SPT	SCT	HLB	IPS
SPD	**0.761**				
SPT	−0.488^***^	**0.719**			
SCT	−0.472^***^	0.556^***^	**0.762**		
HLB	−0.487^***^	0.667^***^	0.527^***^	**0.799**	
IPS	−0.243^***^	0.231^***^	0.263^***^	0.281^***^	**0.812**

SPD, smartphone dependence; SPT, sport participation; SCT, self-control; HLB, health beliefs; IPS, interpersonal support. Values on the diagonal in bold indicate the square roots of average variance extracted (AVE) for each construct. Discriminant validity is supported when the square root of AVE for each construct exceeds its correlations with other constructs. ^***^p < 0.001.

### 3.2 Common method bias assessment

To examine the potential impact of common method bias (CMB), both exploratory and confirmatory approaches were employed. First, Harman’s single-factor test was conducted via exploratory factor analysis (EFA). The unrotated solution extracted 19 factors with eigenvalues greater than 1, and the first factor accounted for 27.381% of the total variance, which is below the commonly used threshold of 40%, suggesting that no single factor dominated the variance structure.

To further assess CMB, confirmatory factor analyses (CFA) were conducted to compare three competing models: a one-factor model, a five-factor model corresponding to the hypothesized latent constructs, and a six-factor model incorporating a latent method factor in addition to the five substantive factors. As shown in Table 4, the one-factor model exhibited poor fit (χ^2^/df = 21.957, CFI = 0.749, TLI = 0.715, SRMR = 0.082, RMSEA = 0.114), indicating that a single latent factor could not account for the observed covariance among items. In contrast, the five-factor model demonstrated excellent fit (CFI = 0.976, TLI = 0.971, SRMR = 0.035, RMSEA = 0.036), and the six-factor model showed only marginal improvement (CFI = 0.980, TLI = 0.977, SRMR = 0.031, RMSEA = 0.032). The χ^2^ difference tests confirmed that both the five- and six-factor models fit the data significantly better than the one-factor solution (*p* < 0.05), but the added method factor did not substantially enhance model performance. These findings, together with the EFA results, suggest that common method bias is not a serious concern in the present study.

**Table 4:** Model fit indices from confirmatory factor analyses for common method bias assessment.

Model	*χ* ^2^	df	*χ*^2^/df	CFI	TLI	SRMR	RMSEA (90% CI)
One-factor model	2964.219	135	21.957	0.749	0.715	0.082	0.114 (0.111–0.118)
Five-factor model	391.952	125	3.136	0.976	0.971	0.035	0.036 (0.032–0.041)
Six-factor model	331.579	124	2.674	0.980	0.977	0.031	0.032 (0.028–0.036)

The one-factor model specifies all items loading onto a single latent factor, representing a common method. The five-factor model specifies items loading onto their respective theoretical constructs. The six-factor model adds a latent method factor to the five theoretical constructs. CFI, comparative fit index; TLI, Tucker–Lewis index; SRMR, standardized root mean square residual; RMSEA, root mean square error of approximation; CI, confidence interval. *χ*^2^ difference tests showed that both the five- and six-factor models fit the data significantly better than the one-factor model (*p* < 0.05).

### 3.3 Group differences in key variables

Descriptive statistics and group comparisons across demographic variables are presented in Table 5.

**Table 5:** Descriptive statistics and differences across demographic variables for key variables.

Demographic variables	Category	SPD		SPT		SCT		HLB		IPS	
		M	SD	M	SD	M	SD	M	SD	M	SD
Gender	Male	2.37	0.76	3.55	0.80	3.63	0.68	3.49	0.79	2.96	0.73
	Female	2.49	0.77	3.54	0.80	3.54	0.70	3.62	0.77	3.18	0.72
	t	−2.996^**^		0.149		2.740^**^		−3.179^**^		−6.003^***^	
	Cohen’s d	−0.149		0.007		0.137		−0.158		−0.299	
Grade	Grade 5	2.77	0.76	3.43	0.80	3.45	0.67	3.58	0.78	3.04	0.70
	Grade 6	2.44	0.75	3.56	0.82	3.63	0.71	3.61	0.80	3.14	0.73
	Grade 7	2.42	0.75	3.53	0.78	3.56	0.71	3.51	0.77	2.99	0.75
	Grade 8	2.31	0.73	3.60	0.79	3.65	0.67	3.56	0.76	3.14	0.75
	Grade 9	1.98	0.62	3.70	0.78	3.70	0.69	3.49	0.82	3.05	0.72
	F	32.825^***^		3.450^**^		5.136^***^		1.281		3.071^*^	
	η^2^	0.076		0.009		0.013		0.003		0.008	
School location	Urban	2.42	0.75	3.56	0.79	3.59	0.69	3.55	0.78	3.04	0.74
	Rural	2.44	0.78	3.54	0.81	3.58	0.70	3.57	0.79	3.11	0.73
	t	−0.492		0.505		0.429		−0.568		−1.894	
	Cohen’s d	−0.025		0.025		0.021		−0.028		−0.094	
Sport Type	Dual sports	2.46	0.78	3.53	0.80	3.57	0.68	3.59	0.75	3.05	0.73
	Team sports	2.44	0.76	3.54	0.78	3.59	0.69	3.53	0.78	3.06	0.75
	Individual sports	2.35	0.75	3.59	0.84	3.61	0.71	3.56	0.83	3.15	0.69
	F	2.442		0.715		0.259		0.894		1.856	
	η^2^	0.003		0.001		0.000		0.001		0.002	

SPD, smartphone dependence; SPT, sport participation; SCT, self-control; HLB, health beliefs; IPS, interpersonal support. Effect size benchmarks: Cohen’s d (0.20 = small, 0.50 = medium, 0.80 = large); η^2^ (0.01 = small, 0.06 = medium, 0.14 = large). ^*^p < 0.05, ^**^p < 0.01, ^***^p < 0.001.

Significant gender differences emerged in four of the five key variables. Compared with girls, boys reported significantly lower smartphone dependence (*t* = −2.996, *p* < 0.01, *d* = −0.149), higher self-control (*t* = 2.740, *p* < 0.01, *d* = 0.137), lower health beliefs (*t* = −3.179, *p* < 0.01, *d* = −0.158), and lower interpersonal support (*t* = −6.003, *p* < 0.001, *d* = −0.299). No significant gender difference was found for sport participation. Although several gender differences reached statistical significance, the corresponding effect sizes were small.

One-way ANOVA revealed significant grade-level differences for smartphone dependence (*F* = 32.825, *p* < 0.001, η^2^ = 0.076), sport participation (*F* = 3.450, *p* < 0.01, η^2^ = 0.009), self-control (*F* = 5.136, *p* < 0.001, η^2^ = 0.013), and interpersonal support (*F* = 3.071, *p* < 0.05, η^2^ = 0.008). No significant grade-level difference was found for health beliefs. Bonferroni-adjusted *post hoc* comparisons further clarified these grade-level differences. For smartphone dependence, Grade 5 students reported significantly higher scores than students in Grades 6, 7, 8, and 9, all *p* < 0.001. Grade 9 students reported significantly lower smartphone dependence than students in Grades 6, 7, and 8, all *p* < 0.001. Differences among Grades 6, 7, and 8 were not statistically significant. For sport participation, Grade 9 students reported significantly higher scores than Grade 5 students, *p* = 0.007, whereas no other pairwise grade comparisons reached statistical significance. For self-control, Grade 5 students reported significantly lower scores than Grade 6 students, *p* = 0.008, Grade 8 students, *p* = 0.003, and Grade 9 students, *p* = 0.003. Other pairwise grade comparisons for self-control were not statistically significant. For interpersonal support, Grade 6 students reported significantly higher scores than Grade 7 students, *p* = 0.035, whereas no other pairwise grade comparisons were significant. These findings indicate a relatively clear grade-related decrease in smartphone dependence, while grade differences in sport participation, self-control, and interpersonal support were more limited and specific to particular grade comparisons.

No significant differences were observed between urban and rural students on any of the five key variables, and the effect sizes were negligible (|d| < 0.10). Similarly, students engaged in dual, team, or individual sports did not differ significantly across variables, with all *F*-values below the significance threshold and η^2^ values approaching zero. Additional factorial ANOVA analyses tested interaction effects, including gender × grade and school location × sport type, but none reached statistical significance. These findings suggest that group differences were primarily reflected in main effects rather than demographic interaction patterns.

### 3.4 Model fit and path analysis

The hypothesized structural equation model was tested using AMOS 26.0. The model showed good fit to the data: χ^2^(98) = 366.867, χ^2^/df = 3.744, CFI = 0.974, TLI = 0.969, SRMR = 0.038, RMSEA = 0.041, 90% CI [0.037, 0.046]. These fit indices met the recommended cutoffs, supporting the adequacy of the proposed structural model. As specified in the analysis strategy, gender, grade level, school location, and sport type were examined in preliminary group-difference analyses and were not entered as covariates in the SEM. Therefore, the structural model estimated the theory-specified associations among smartphone dependence, self-control, health beliefs, and sport participation without demographic covariate adjustment.

**Figure 2:**
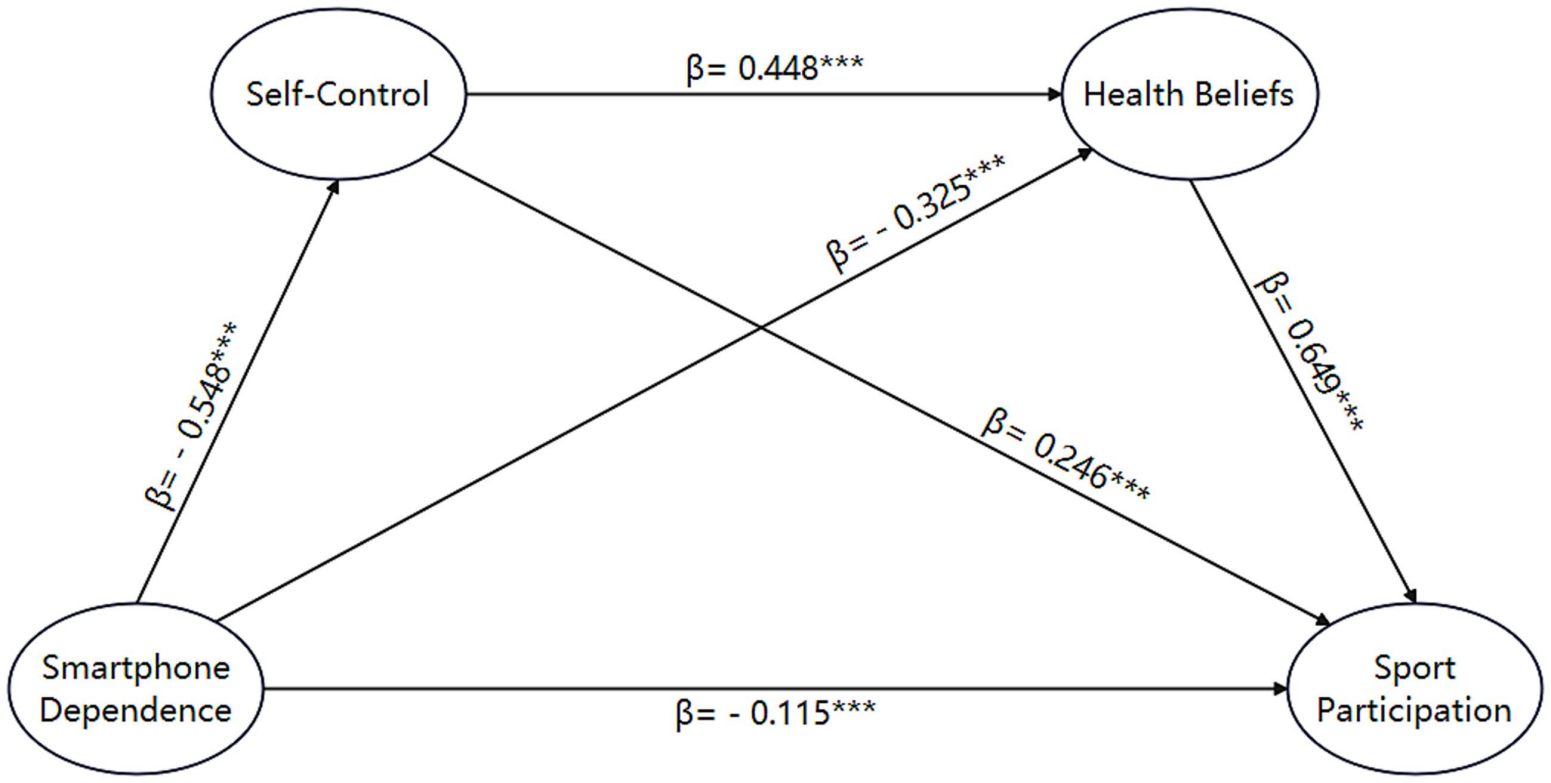
Standardized path coefficients of the structural equation model. Standardized coefficients for the theory-specified mediation model are shown. Gender, grade level, school location, and sport type were examined in preliminary group-difference analyses and were not included as covariates in this SEM. ^***^p < 0.001.

As illustrated in Figure 2, all standardized path coefficients in the structural model were statistically significant at the 0.001 level. Smartphone dependence was negatively associated with self-control, health beliefs, and sport participation. Self-control was positively associated with health beliefs and sport participation, and health beliefs were positively associated with sport participation. These results supported the hypothesized cross-sectional associations among the theoretical variables in the chain mediation model.

### 3.5 Mediation analysis

Bootstrapping procedures with 5,000 resamples were used to examine the significance of direct and indirect associations in the cross-sectional mediation model. As shown in Table 6, the total association between smartphone dependence (SPD) and sport participation (SPT) was significant and negative (*β* = −0.620, 95% CI [−0.670, −0.568]), indicating an overall negative relationship between the two variables.

**Table 6:** Total, direct, and indirect associations in the cross-sectional mediation model.

Path	*β*	Boot SE	Boot LLCI	Boot ULCI	Ratio
Direct association					
SPD → SPT	−0.115^***^	0.034	−0.179	−0.048	18.55%
Indirect associations					
SPD → SCT → SPT	−0.135^***^	0.020	−0.176	−0.098	21.77%
SPD → HLB → SPT	−0.211^***^	0.024	−0.260	−0.166	34.03%
SPD → SCT → HLB → SPT	−0.159^***^	0.015	−0.189	−0.132	25.65%
Total indirect associations	−0.505^***^	0.025	−0.557	−0.459	81.45%
Total association	−0.620^***^	0.026	−0.670	−0.568	100%

SPD, smartphone dependence; SPT, sport participation; SCT, self-control; HLB, health beliefs; β, standardized path coefficient; Boot SE, bootstrap standard error; Boot LLCI, the lower bound of the 95% confidence interval; Boot ULCI, the upper limit of the 95% confidence interval (Percentile Bootstrap Method with Bias Correction). The Bootstrap sample size is set at 5000. ^***^p < 0.001.

The direct association between SPD and SPT remained statistically significant (*β* = −0.115, 95% CI [−0.179, −0.048]), accounting for 18.55% of the total association. In addition, all three specific indirect associations were statistically significant, as their 95% confidence intervals did not include zero. Specifically, SPD was indirectly associated with SPT through self-control alone (SPD → SCT → SPT: *β* = −0.135, 95% CI [−0.176, −0.098], 21.77%), through health beliefs alone (SPD → HLB → SPT: *β* = −0.211, 95% CI [−0.260, −0.166], 34.03%), and through the theoretically specified sequence of self-control and health beliefs (SPD → SCT → HLB → SPT: *β* = −0.159, 95% CI [−0.189, −0.132], 25.65%). The total indirect association was also significant (*β* = −0.505, 95% CI [−0.557, −0.459]), accounting for 81.45% of the total association. These findings indicate that self-control and health beliefs were significant statistical mediators in the cross-sectional association between smartphone dependence and sport participation. The sequential indirect association through self-control and health beliefs should be interpreted as a theory-informed statistical specification rather than evidence of temporal ordering.

### 3.6 Direct-path moderation analysis

To examine whether interpersonal support (IPS) moderated the direct association between smartphone dependence (SPD) and sport participation (SPT), a simple moderation analysis was conducted using the PROCESS macro (Model 1). This analysis tested whether the direct association between SPD and SPT differed across levels of IPS; it did not test whether IPS moderated the indirect associations through self-control or health beliefs. The results are presented in Table 7. The interaction term SPD × IPS was statistically significant (B = −0.362, SE = 0.029, *t* = −12.302, 95% CI [−0.419, −0.304], *p* < 0.001), indicating that the direct association between SPD and SPT differed across levels of IPS. Specifically, the coefficient for SPD was negative and significant (B = −0.492, SE = 0.022, *t* = −22.110, 95% CI [−0.536, −0.449], *p* < 0.001), suggesting that higher smartphone dependence was associated with lower sport participation. The coefficient for IPS was positive and significant (B = 0.135, SE = 0.023, *t* = 5.803, 95% CI [0.089, 0.180], *p* < 0.001), indicating that higher interpersonal support was generally associated with higher sport participation. The significant negative interaction coefficient further indicated that the negative association between SPD and SPT was more pronounced among adolescents reporting higher levels of IPS.

**Table 7:** Simple moderation analysis of interpersonal support on the direct association between smartphone dependence and sport participation.

Predictor	SPT				
	B	SE	t	LLCI	ULCI
Constant	3.497^***^	0.017	206.082	3.464	3.531
SPD	−0.492^***^	0.022	−22.11	−0.536	−0.449
IPS	0.135^***^	0.023	5.803	0.089	0.180
SPD * IPS	−0.362^***^	0.029	−12.302	−0.419	−0.304

SPD, smartphone dependence; SPT, sport participation; IPS, interpersonal support; B, unstandardized regression coefficient; SE, standard error of B; t, t-value for the significance test of the coefficient; LLCI, lower bound of the 95% confidence interval; ULCI, upper bound of the 95% confidence interval. This model tested simple moderation of the direct association between SPD and SPT. Conditional indirect effects were not estimated. ^***^p < 0.001.

**Figure 3:**
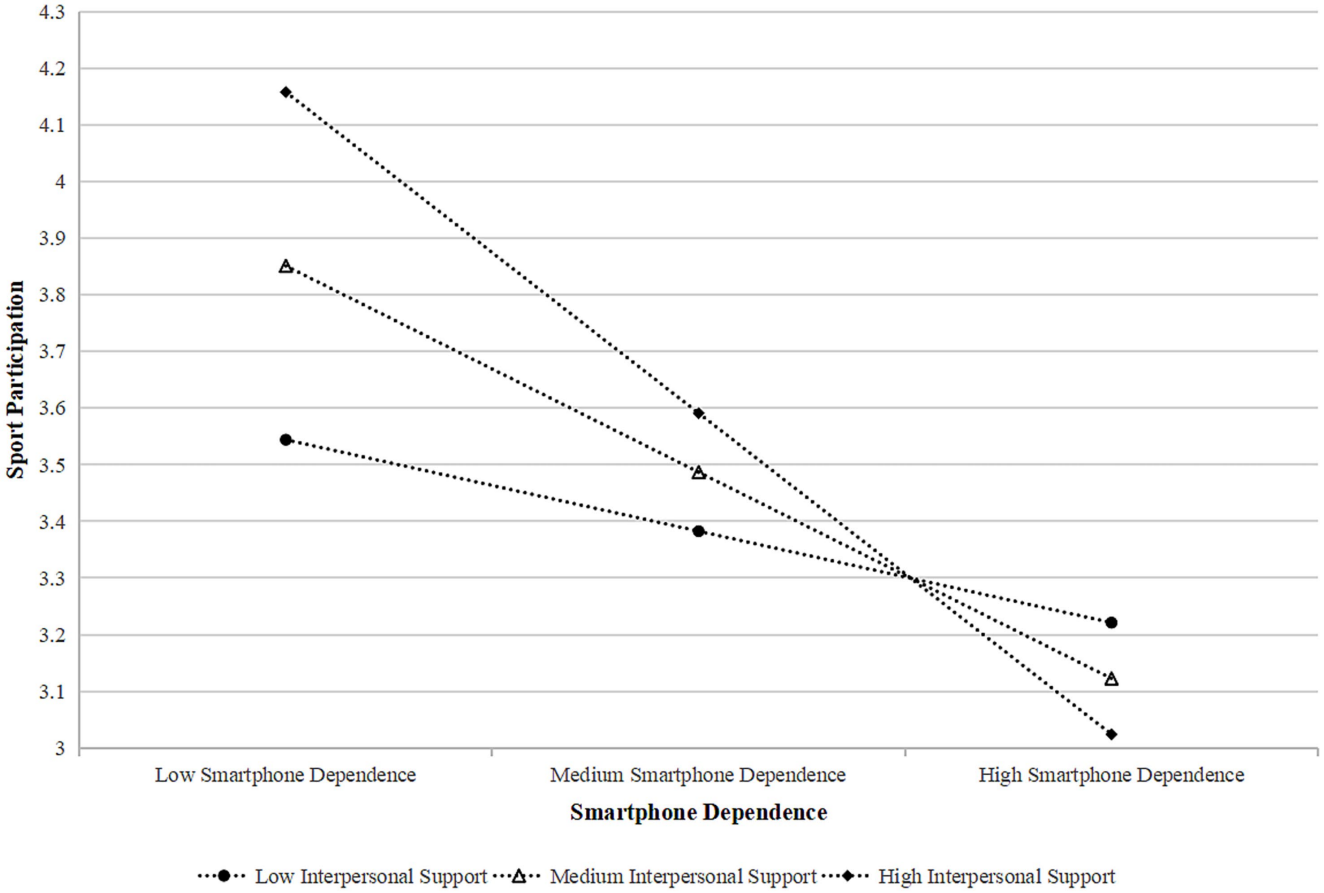
Interaction effect of smartphone dependence and interpersonal support on sport participation.

As illustrated in Figure 3, the negative association between SPD and SPT was steeper at higher levels of IPS. Adolescents reporting higher interpersonal support showed a stronger negative association between smartphone dependence and sport participation, whereas those reporting lower interpersonal support showed a relatively weaker negative association. This pattern suggests a strengthening rather than buffering interaction in the present sample. However, this result should not be interpreted as evidence that interpersonal support is harmful. Rather, it suggests that general perceived support may not necessarily function as sport-specific support in the context of smartphone dependence. Because the IPS measure assessed general support from family members, friends/peers, and significant others, and did not separately distinguish teacher-specific, sport-specific, online/offline, emotional, or instrumental support, the moderation finding should be interpreted cautiously.

## 4 Discussion

### 4.1 Demographic differences in key variables

The observed demographic differences across gender and grade provide a descriptive overview of adolescents’ behavioral and psychological patterns related to smartphone use and sport participation. Boys reported significantly higher self-control than girls, whereas girls reported higher levels of smartphone dependence, health beliefs, and interpersonal support. These gender-related differences can be interpreted in light of broader developmental and socialization perspectives. Previous research has suggested that gender differences in temperament and socioemotional tendencies may be related to differences in behavioral regulation, relational orientation, and emotional expressivity (Else-Quest et al., 2006). In this context, the higher interpersonal support and health beliefs reported by girls in the present study may reflect a more relationally oriented pattern of self-perception and health-related cognition, whereas their higher smartphone dependence may be associated with stronger digital engagement in interpersonal or emotional contexts. However, these interpretations should be considered descriptive and tentative because of the cross-sectional nature of the data and the small effect sizes observed for gender differences.

Smartphone dependence showed significant grade-level differences, with the clearest contrast observed between Grade 5 and the higher grades, and between Grade 9 and several lower grades. This pattern suggests that smartphone dependence was lower among older students in the present sample. Self-control and sport participation also differed across grades, but *post hoc* comparisons indicated that these differences were more limited and specific to particular grade pairs. This grade-related pattern is broadly compatible with developmental evidence suggesting that executive functions continue to mature during adolescence, including capacities related to impulse control, self-monitoring, and goal-directed behavior (Best and Miller, 2010). The observed differences in self-control and sport participation are also consistent with longitudinal research reporting developmental links between sport participation and self-control ability during adolescence (Chen et al., 2025). Nevertheless, the present findings should be interpreted as grade-related differences observed within a cross-sectional sample, rather than as evidence of developmental change over time.

Interpersonal support also differed significantly across grades, although the pattern was not linear. *Post hoc* comparisons showed that the clearest difference was between Grades 6 and 7. This non-linear pattern may be understood in relation to the changing structure of adolescents’ social relationships during school transitions and early-to-middle adolescence. Prior developmental research suggests that adolescence is characterized by shifts in family, peer, and broader interpersonal relationships, including increasing autonomy and changing relational expectations (Laursen and Collins, 2009; Orben et al., 2020; Niedtfeld et al., 2025). In the present study, however, the data do not allow identification of the specific sources of support that contributed to these grade-level differences. Therefore, this pattern should be interpreted cautiously as descriptive variation in perceived interpersonal support across grades.

In contrast, neither school location nor sport type showed statistically significant differences across the five key variables. In the present sample, urban and rural students reported broadly comparable levels of smartphone dependence, sport participation, self-control, health beliefs, and interpersonal support. Although previous research has documented rural–urban educational inequalities and spatial variations in China (Xiang and Stillwell, 2023), such contextual distinctions were not clearly reflected in these psychosocial and behavioral variables within the present sample. Similarly, the absence of significant differences across individual, dual, and team sports suggests that broad sport categories may not be sufficiently sensitive to capture variation in adolescents’ psychological characteristics or participation experiences. Future studies could examine more detailed sport-related indicators, such as participation frequency, training context, competitive level, competitive versus recreational participation, and motivational orientation (Li et al., 2024).

Finally, although interpersonal support showed a significant conditional association in the relationship between smartphone dependence and sport participation, additional demographic interaction analyses did not reveal significant interaction patterns involving gender, grade, school location, or sport type. This suggests that the demographic variables examined in the present study were mainly related to differences in mean levels of the key constructs, rather than to clearly differentiated patterns of association among these variables. However, because the present analyses were not designed as formal multi-group moderation or moderated mediation tests, conclusions about subgroup differences should remain cautious. Future research could use longitudinal, multi-group, or covariate-adjusted models to examine whether the associations among smartphone dependence, interpersonal support, and sport participation vary across demographic subgroups.

### 4.2 Indirect psychological associations between smartphone dependence and sport participation

The present findings indicate that self-control and health beliefs were significant statistical mediators in the cross-sectional association between smartphone dependence and sport participation among adolescents. Rather than suggesting a causal mechanism, these results are consistent with the broader view that physical activity engagement is associated with a constellation of cognitive, motivational, and self-regulatory factors, in addition to situational constraints (Baumeister et al., 2007; Hagger and Chatzisarantis, 2009). In this section, mediation terminology refers to statistical indirect associations within the proposed theoretical model, not to temporally verified causal processes.

First, self-control represented a significant indirect association between smartphone dependence and sport participation. This pattern is theoretically consistent with dual-systems models of self-regulation, which emphasize the relevance of inhibitory control, sustained attention, and goal maintenance in managing conflicts between immediate gratification and long-term goals (Hofmann et al., 2012). In the present study, adolescents with higher smartphone dependence tended to report lower self-control, and lower self-control was associated with lower sport participation. Because regular sport participation usually involves routine commitment, delayed gratification, and sustained engagement, self-control may be an important psychological correlate of adolescents’ participation in physically active lifestyles. This interpretation is also consistent with longitudinal evidence reporting negative associations among problematic smartphone use, self-control, and physical activity among adolescents (Zhao et al., 2024).

Second, health beliefs showed the largest single indirect association among the examined indirect associations. This finding suggests that adolescents’ health-related cognitions may be particularly relevant to the association between smartphone dependence and sport participation. From the perspective of the Health Belief Model, health beliefs reflect individuals’ perceptions of health risks, the seriousness of possible negative outcomes, the benefits of health-related behaviors, and confidence in their ability to act (Rosenstock, 1974). In the present study, higher smartphone dependence was associated with weaker health beliefs, which were in turn associated with lower sport participation. This pattern may indicate that adolescents with weaker sport-related health beliefs are less likely to perceive sport participation as personally meaningful, beneficial, or necessary. Such an interpretation is particularly relevant during adolescence, when health-related cognition and behavioral preferences are still developing and may be embedded in broader media and social contexts (Wang et al., 2025).

The sequential indirect association from smartphone dependence through self-control and health beliefs to sport participation was also significant. This finding suggests that self-control and health beliefs may represent interrelated psychological correlates in the association between smartphone dependence and sport participation. The theoretically specified sequence positioned self-control before health beliefs because self-control reflects a broader regulatory capacity that may be relevant to attention to long-term goals, resistance to immediately rewarding digital engagement, and maintenance of value-guided behavior. Health beliefs were positioned as more proximal to sport participation because they reflect adolescents’ evaluations of the value, necessity, benefits, and feasibility of engaging in sport. This interpretation is consistent with self-control theory, which emphasizes the relevance of self-regulatory resources to long-term goals and value-guided behavior (Baumeister et al., 1998), and with perspectives suggesting that self-regulatory processes are closely linked to broader motivational and cognitive systems (Inzlicht and Schmeichel, 2012).

At the same time, the cross-sectional design does not allow the proposed sequence to be interpreted as evidence of temporal ordering. Alternative or reciprocal patterns remain possible. For example, adolescents with stronger sport-related health beliefs may also be more motivated to regulate smartphone use and maintain active routines. Likewise, adolescents who participate more frequently in sport may develop stronger self-regulatory habits and more favorable health beliefs over time. Therefore, the sequential association observed in the present study should be understood as one theory-informed statistical specification rather than the only possible ordering among these variables. Future longitudinal, diary-based, or cross-lagged studies are needed to compare alternative temporal models and examine whether the proposed sequence is stable over time.

Taken together, these findings suggest that the association between smartphone dependence and sport participation is reflected not only in a direct statistical association, but also in indirect associations involving self-control and health beliefs. The relatively large proportion of the total association accounted for by indirect associations suggests that psychological and motivational variables may be important for understanding differences in adolescents’ sport participation. From an applied perspective, these results may inform hypotheses for future longitudinal and intervention studies examining whether school-based health education, self-regulation components, and sport-related health belief education are prospectively associated with later sport participation.

### 4.3 Conditional role of interpersonal support

The present findings indicate that interpersonal support was a significant conditional factor in the direct association between smartphone dependence and sport participation. Specifically, the negative association between smartphone dependence and sport participation was stronger among adolescents reporting higher levels of perceived interpersonal support. Although this pattern may appear counterintuitive, it highlights the complexity of social-contextual factors and suggests that interpersonal support may have different behavioral meanings depending on adolescents’ level of smartphone dependence and the contexts in which support is experienced.

Interpersonal support is often discussed as a positive psychosocial resource associated with psychological resilience and health-related behaviors (Cohen and Wills, 1985; Southwick et al., 2016). Previous studies have also reported that adolescents with higher perceived support from parents, peers, or teachers tend to show higher levels of physical activity (Dong et al., 2025; Dishman et al., 2009). In the present study, interpersonal support was positively associated with sport participation overall. However, the significant interaction between interpersonal support and smartphone dependence suggests that the negative association between smartphone dependence and sport participation was more pronounced among adolescents with higher perceived interpersonal support. This moderation result should be interpreted as a direct-path interaction rather than as evidence that interpersonal support moderated the indirect associations through self-control or health beliefs.

One possible interpretation is that adolescents with higher interpersonal support may have broader social connections that are partly maintained through digital channels, such as messaging apps and social media platforms. When perceived support is closely tied to smartphone-based communication, supportive social networks may also be associated with more frequent digital interaction. In this context, higher interpersonal support may coexist with peer norms or social expectations surrounding mobile phone use, which may be related to more intensive smartphone-based social engagement. This interpretation is consistent with evidence that peer pressure surrounding mobile phone use is associated with adolescents’ mobile social media addiction, as adolescents may engage more frequently in smartphone-based interaction to maintain peer relationships and conform to social norms (Xu et al., 2023). In such cases, interpersonal support may be associated with emotional connectedness while also being linked to more screen-based forms of social participation.

Additionally, the interaction pattern may be understood from a “resource misalignment” perspective. High perceived psychosocial support may not necessarily correspond to action-oriented or sport-specific support. Adolescents may feel emotionally supported, but such support may not be directly related to encouragement, companionship, or accountability for sport participation unless it is explicitly connected to physical activity contexts. This explanation is consistent with the conceptual distinction between emotional and instrumental support (Thoits, 2011). From this perspective, general interpersonal support may not be sufficient to offset the lower sport participation associated with higher smartphone dependence when that support is not oriented toward active lifestyles.

Therefore, the present finding should not be interpreted as evidence that interpersonal support is detrimental or harmful. Rather, it suggests that the behavioral meaning of general perceived support may depend on whether support is enacted through digital interaction, emotional reassurance, instrumental assistance, or concrete sport-related encouragement. This distinction is particularly important because the IPS measure used in the present study assessed general perceived support from family members, friends/peers, and significant others, but did not separately identify teacher-specific, sport-specific, online/offline, emotional, or instrumental forms of support. Teacher support may be especially relevant in school-based sport participation because teachers can provide physical education guidance, digital-use guidance, outdoor activity opportunities, and structured encouragement for active lifestyles. Future studies should therefore distinguish general interpersonal support from support that is specifically oriented toward sport participation and active living.

From a practical perspective, these findings suggest that interpersonal support should not be assumed to be uniformly protective in all behavioral contexts. Its relevance may depend on how support is enacted and whether it is connected to active, goal-oriented behaviors. Future school-based programs concerned with adolescent sport participation could test whether general interpersonal support can be translated into sport-specific support, such as shared activity, peer encouragement for exercise, teacher guidance in physical education, family involvement, goal scaffolding, and accountability structures, rather than relying only on emotional reassurance.

### 4.4 Theoretical implications

The present study offers several theoretical implications for understanding adolescent sport participation in the context of smartphone dependence. First, by integrating self-control and health beliefs into a chain mediation model, this study provides preliminary cross-sectional evidence that explanations of digital technology use and health-related behavior may extend beyond a simple displacement perspective. The findings suggest that the association between smartphone dependence and sport participation is not only reflected in reduced time or opportunity for physical activity, but is also statistically linked to adolescents’ self-regulatory and health-related cognitive resources. In this sense, the study contributes to a more psychologically grounded understanding of how digital dependence is associated with physical activity behavior during adolescence.

Second, the significant sequential indirect association from smartphone dependence to sport participation through self-control and health beliefs highlights the potential interconnection between regulatory capacity and health-related cognition. This finding suggests that self-control and health beliefs may be usefully considered together rather than as isolated psychological variables. Adolescents’ ability to regulate impulses and maintain goal-directed behavior may be closely associated with how they perceive the value, necessity, and benefits of sport participation. However, because the proposed sequence was tested using cross-sectional data, it should be viewed as a theoretically specified statistical model rather than evidence of a confirmed developmental process. This provides a useful framework for future research examining how digital behavior, executive functioning, health cognition, and physical activity are jointly embedded in adolescent development.

Third, the conditional role of interpersonal support adds a social-contextual dimension to the model. Although interpersonal support is often regarded as a positive psychosocial resource, the present study found that the negative association between smartphone dependence and sport participation was stronger among adolescents with higher perceived interpersonal support. This pattern suggests that social support may have different behavioral meanings depending on how it is enacted and whether it is connected to digital communication or active lifestyles. Importantly, the present study tested direct-path moderation rather than moderated mediation; thus, the findings should not be interpreted as evidence that interpersonal support changes the indirect associations through self-control or health beliefs. Instead, this result contributes to a more nuanced understanding of interpersonal support by distinguishing general perceived support from support that is specifically oriented toward sport participation and active living.

### 4.5 Practical implications

Because of the cross-sectional design, the present findings should not be interpreted as evidence that changing self-control, health beliefs, or interpersonal support will necessarily increase sport participation. Instead, the findings provide preliminary targets and hypotheses for future longitudinal and intervention research on adolescent sport participation and digital wellbeing.

First, future school-based intervention development may consider more than screen-time reduction alone. The significant statistical indirect associations involving self-control and health beliefs suggest that adolescents’ sport participation is closely related to self-regulatory skills, such as goal setting, time management, impulse control, and planning for regular physical activity. Health education components could also be designed and tested to address the personal relevance and perceived benefits of sport participation, so that physical activity is understood not merely as a school requirement but as a meaningful component of daily wellbeing. However, the effectiveness of such components should be evaluated in longitudinal or experimental designs.

Second, the findings point to the importance of distinguishing general interpersonal support from sport-specific and action-oriented support. Although interpersonal support was positively associated with sport participation overall, the interaction pattern showed that higher general support did not necessarily weaken the negative association between smartphone dependence and sport participation. Therefore, future programs could examine whether parents, teachers, and peers can provide more concrete forms of support, such as exercising together, reminding students of activity goals, creating opportunities for participation, encouraging offline peer activity, and reinforcing active rather than screen-based forms of social connection. In particular, teacher support may be important in school contexts because teachers and physical education staff can help translate general encouragement into structured opportunities for sport participation.

Third, school-based strategies may need to be sensitive to grade-related differences, although these differences should not be interpreted as developmental trends based on the present cross-sectional data. The patterns observed in this study suggest that smartphone dependence, self-control, sport participation, and interpersonal support vary across school stages. Future intervention development could explore whether younger students benefit more from basic self-regulation habits and awareness of passive screen-based routines, whereas older students may benefit more from autonomous motivation, health responsibility, peer support for active lifestyles, and strategies for balancing digital communication with physical activity. Such differentiated strategies should be tested empirically before being adopted as evidence-based recommendations.

### 4.6 Limitations and future directions

Several limitations should be considered when interpreting the findings of this study. First, the cross-sectional design limits conclusions about the temporal ordering among smartphone dependence, self-control, health beliefs, interpersonal support, and sport participation. Although the proposed chain mediation model showed acceptable model fit and the indirect associations through self-control and health beliefs were statistically significant, the present data cannot determine whether higher smartphone dependence is followed by lower self-control and weaker health beliefs, or whether adolescents with lower sport participation and weaker self-regulatory resources are more likely to report higher smartphone dependence. This issue is particularly important for the sequential association identified in this study, because the ordering from smartphone dependence to self-control, from self-control to health beliefs, and from health beliefs to sport participation was theoretically specified rather than temporally observed. Similarly, the conditional role of interpersonal support should be interpreted as a statistical interaction within the present sample, rather than evidence that interpersonal support changes the association between smartphone dependence and sport participation over time. Future studies could use longitudinal, diary-based, experimental, or cross-lagged panel designs to examine whether the observed associations remain stable across time and whether reciprocal patterns exist among digital media use, self-regulation, health-related beliefs, and sport participation.

Second, all key variables were assessed using self-report questionnaires. Although the measures demonstrated acceptable reliability and measurement validity in the present adolescent sample, self-reported smartphone dependence, sport participation, self-control, health beliefs, and interpersonal support may still be influenced by recall bias, social desirability, and shared response tendencies. This limitation is especially relevant because the model tested in this study connects several psychological and behavioral variables reported by the same students at the same time point. For example, students who perceive themselves as more self-controlled may also be more likely to report stronger health beliefs and higher sport participation, which may partly reflect a general positive self-evaluation tendency. Although common method bias was examined using both exploratory and confirmatory approaches, these analyses cannot fully rule out shared method variance. Future research could strengthen measurement by combining self-report data with smartphone usage records, wearable or app-based activity indicators, school records of sport participation, teacher or parent ratings of self-control, and peer or family reports of interpersonal support.

Third, although reliability analysis and CFA supported the measurement quality of the instruments in the present sample, several measures were established or adapted from instruments developed in related but not identical populations. For example, some scales were originally developed or validated among older adolescents, youths, or college students, and the response format of interpersonal support was adapted from a 7-point to a 5-point format to improve consistency and comprehensibility for younger participants. Although this adaptation was appropriate for the present school-based survey, future research should conduct more extensive age-specific validation, including cognitive interviews, pilot testing, item comprehension checks, and measurement invariance analyses across grade levels, gender groups, and school contexts.

Fourth, the measurement of sport participation and smartphone dependence could be further refined. In the present study, sport participation was assessed through self-reported intensity, frequency, and duration, which provided an efficient overall indicator of adolescents’ participation level. However, this approach did not distinguish between different forms of sport participation, such as physical education classes, school-organized sport, informal peer activity, competitive training, and leisure-time exercise. These forms of participation may involve different levels of autonomy, peer involvement, teacher support, and self-regulatory demand. Similarly, smartphone dependence was measured as a general dependence-related tendency, including loss of control, withdrawal, avoidance, and inefficiency, but the measure did not distinguish among entertainment-based use, learning-related use, social communication, gaming, or short-video consumption. This distinction is important for the present model because different smartphone-use purposes may be differently related to self-control, health beliefs, interpersonal support, and sport participation. Future studies could examine whether the indirect associations identified in this study vary according to specific types of smartphone engagement and specific forms of sport participation.

Fifth, interpersonal support was measured as a general perceived support construct. This was appropriate for examining adolescents’ broader social resources, but it limited the interpretation of the unexpected moderation pattern found in this study. In the present results, interpersonal support was positively associated with sport participation overall, yet the negative association between smartphone dependence and sport participation was stronger among adolescents with higher perceived interpersonal support. One possible reason is that the measure did not distinguish between support that is emotionally reassuring, digitally mediated, peer-based, family-based, teacher-based, offline, instrumental, or specifically oriented toward sport participation. Teacher support was not measured as a separate IPS dimension in the present study. This is important because teachers may shape adolescents’ sport participation through physical education instruction, digital-use guidance, outdoor activity organization, and encouragement of active lifestyles. Future research should therefore distinguish general interpersonal support from sport-specific support, such as parental encouragement for exercise, peer companionship in sport, teacher support in physical education, and support delivered through offline versus digital contexts. Such distinctions would help clarify whether the conditional pattern observed in this study reflects the nature, source, or behavioral direction of interpersonal support.

Sixth, although the sample included Chinese adolescents from Grades 5–9 and covered different genders, grade levels, school locations, and sport types, the findings should be interpreted with attention to the school-based sampling context. Participants were recruited from approximately 40 schools in four cities of Shandong Province using a stratified school-based convenience sampling approach. Therefore, the sample should not be interpreted as nationally representative of all Chinese adolescents. In addition, although the sampling procedure was school-based, school and class identifiers were not retained in the analytic dataset; therefore, cluster-adjusted standard errors could not be estimated. Future studies should use multilevel or cluster-robust approaches to account for the nested structure of students within classes and schools. Relatedly, gender, grade level, school location, and sport type were examined in preliminary group-difference analyses but were not included as covariates in the SEM or moderation model. Future research could test whether the present associations remain robust in covariate-adjusted, multilevel, or multi-group models.

Seventh, this study examined two psychological mediators and one social-contextual moderator, but other relevant variables were not included. The chain mediation model focused on self-control and health beliefs because they represent self-regulatory and health-cognitive correlates of sport participation. However, adolescents’ sport participation in the context of smartphone dependence may also be related to academic pressure, sleep quality, parental monitoring, peer norms regarding smartphone use, school sport climate, access to facilities, physical education quality, and students’ future orientation. These variables may be especially important for interpreting the indirect association from self-control to health beliefs and then to sport participation. For example, students with stronger future orientation may be more likely to value long-term health benefits, regulate smartphone use, and maintain sport routines (Chen and Vazsonyi, 2013), whereas students under high academic pressure or with limited school sport opportunities may find it harder to translate health beliefs into actual participation. In particular, the current measure of smartphone dependence did not distinguish between entertainment-based, learning-related, and socially oriented use, which may be relevant to the conditional role of interpersonal support (Mei et al., 2024). Future studies could incorporate these contextual and behavioral variables to examine whether the associations identified in the present model remain robust when broader school, family, peer, and lifestyle factors are considered.

Finally, the present study used a variable-centered analytic approach based on mediation and moderation analyses. This approach was useful for examining the overall pattern of associations among smartphone dependence, self-control, health beliefs, interpersonal support, and sport participation. However, adolescents may not all fit the same psychosocial pattern. For example, some students may report relatively high smartphone dependence but also maintain high sport participation, possibly because they have stronger sport-specific support or established sport routines. Others may report low sport participation together with weaker self-control, weaker health beliefs, and limited sport-oriented support. Future research could use person-centered approaches, such as latent profile analysis, latent class analysis, or configurational methods such as fsQCA, to identify subgroups of adolescents with different combinations of digital behavior, psychological resources, social support, and sport participation. Such work would provide more differentiated evidence for understanding which adolescents may be more likely to show lower sport participation in the context of smartphone dependence and which forms of psychological or social support may be most relevant for different groups.

## 5 Conclusion

In conclusion, this cross-sectional study found that smartphone dependence was negatively associated with sport participation among Chinese adolescents, and that this association was statistically reflected in indirect associations involving self-control, health beliefs, and their theoretically specified sequence. Adolescents reporting higher smartphone dependence tended to report lower self-control and weaker sport-related health beliefs, which were in turn associated with lower sport participation. Interpersonal support showed a more complex direct-path moderation pattern: although it was positively associated with sport participation overall, the negative association between smartphone dependence and sport participation was more pronounced among adolescents with higher perceived interpersonal support. This finding should not be interpreted as evidence that interpersonal support is harmful; rather, it suggests that general perceived support may not necessarily function as sport-specific or action-oriented support in the context of smartphone dependence. Taken together, the findings highlight the relevance of self-regulatory capacity, sport-related health cognition, and the specific nature of social support when understanding adolescents’ sport participation in digitally saturated school and social contexts. Given the cross-sectional design, these results should be interpreted as theory-informed statistical associations rather than causal or temporal processes. They may provide preliminary guidance for future longitudinal and intervention research examining whether smartphone-use awareness, self-control development, sport-related health belief education, and action-oriented social support are prospectively associated with adolescent sport participation.

## Data Availability

The original contributions presented in the study are included in the article/Supplementary material, further inquiries can be directed to the corresponding author.
